# Firefly Algorithm for Cardinality Constrained Mean-Variance Portfolio Optimization Problem with Entropy Diversity Constraint

**DOI:** 10.1155/2014/721521

**Published:** 2014-05-29

**Authors:** Nebojsa Bacanin, Milan Tuba

**Affiliations:** Faculty of Computer Science, Megatrend University Belgrade, 11070 Belgrade, Serbia

## Abstract

Portfolio optimization (selection) problem is an important and hard optimization problem that, with the addition of necessary realistic constraints, becomes computationally intractable. Nature-inspired metaheuristics are appropriate for solving such problems; however, literature review shows that there are very few applications of nature-inspired metaheuristics to portfolio optimization problem. This is especially true for swarm intelligence algorithms which represent the newer branch of nature-inspired algorithms. No application of any swarm intelligence metaheuristics to cardinality constrained mean-variance (CCMV) portfolio problem with entropy constraint was found in the literature. This paper introduces modified firefly algorithm (FA) for the CCMV portfolio model with entropy constraint. Firefly algorithm is one of the latest, very successful swarm intelligence algorithm; however, it exhibits some deficiencies when applied to constrained problems. To overcome lack of exploration power during early iterations, we modified the algorithm and tested it on standard portfolio benchmark data sets used in the literature. Our proposed modified firefly algorithm proved to be better than other state-of-the-art algorithms, while introduction of entropy diversity constraint further improved results.

## 1. Introduction

Since most real-life problems can be modeled as optimization tasks, many methods and techniques that could tackle such problems were developed. Thus, the optimization became one of the most applicable fields in mathematics and computer science.

The difficulty of an optimization problem depends on the mathematical relationships between the objective function, potential constraints, and decision variables. Hard optimization problems can be combinatorial (discrete) or continuous (global optimization), while continuous problems can be further be classified as constrained or unconstrained (bound constrained).

The optimization problem becomes even harder when some variables are real-valued, while others can take only integer values. Such mixed continuous/discrete problems usually require problem-specific search techniques in order to generate optimal, or near optimal solution.

One representative example of such hard optimization problems is portfolio optimization, a well-known issue in economics and finance. Many methods and heuristics were developed to optimize various models and formulations of the portfolio problem [1]. Various portfolio optimization problem models may or may not include different constraints in their formulations. Also, to enhance the diversity of portfolio, some approaches include entropy in its formulations [2].

Unconstrained (bound constrained) optimization is formulated as *D*-dimensional minimization or maximization problem:

(1) $$\begin{array}{c} \min\;\left(\text{or}\;\max\right)f\left(x\right),\;x=\left(x_{1},x_{2},x_{3},\ldots ,x_{D}\right)\in S, \end{array}$$

where *x* represents a real vector with *D* ≥ 1 components and *S* ∈ *R* ^*D*^ is hyper-rectangular search space with *D* dimensions constrained by lower and upper bounds:

(2) $$\begin{array}{c} lb_{i}\leq x_{i}\leq ub_{i},\;i\in \left[1,D\right]. \end{array}$$

In (2), *lb*_*i*_ and *ub*_*i*_ are lower and upper bounds for the *i*th problem component, respectively.

The nonlinear constrained optimization problem in the continuous space can be formulated in the same way as in (1), but in this case *x* ∈ *F*⊆*S*, where *S* is *D*-dimensional hyper-rectangular space as defined in (2) while *F*⊆*S* represents the feasible region defined by the set of *m* linear or nonlinear constraints:

(3) $$\begin{array}{c} g_{j}\left(x\right)\leq 0,\;\text{for}\;\;j\in \left[1,q\right],\\ h_{j}\left(x\right)=0,\;\text{for}\;\;j\in \left[q+1,m\right], \end{array}$$

where *q* is the number of inequality constraints, and *m* − *q* is the number of equality constraints.

Fundamental versions of algorithms and metaheuristics for constrained numerical optimization problems do not include methods for dealing with constraints. Therefore, constraint handling techniques are usually added to these algorithms to improve and redirect the search process towards the feasible region of the search space. Equality constraints make optimization even harder by shrinking the feasible search space which becomes very small compared to the entire search space. To tackle such problem, equality constraints are replaced with the inequality constraints [3]:

(4) $$\begin{array}{c} |h\left(x\right)|-\upsilon \leq 0, \end{array}$$

where *υ* > 0 is some small violation tolerance, usually dynamically adjusted.

Since hard optimization problems are unsolvable in a reasonable amount of time, the exact methods which trace optimal solution cannot be applied. For such problems, it is more appropriate to employ nondeterministic metaheuristics.

Metaheuristics are iterative, population based, and stochastic approaches that do not guarantee finding the optimal solution, but they can obtain satisfying solution within acceptable time [4]. In metaheuristics implementations, the processes of exploitation and exploration conduct the search process. Exploitation directs search around the current best solutions, while the exploration randomly discovers new regions of a search domain.

During the last few decades, we witnessed development of nature-inspired sophisticated intelligent systems that can be used as optimization tools for many complex and hard problems. Metaheuristics that incorporate and simulate natural principles and rules are called nature-inspired metaheuristics.

Nature-inspired metaheuristics [5] can roughly be divided into two categories: evolutionary algorithms (EA) and swarm intelligence. The most prominent representative of EA is genetic algorithms (GA). GA can obtain good results for many kinds of optimization problems [6].

Social behavior of swarms of ants, bees, worms, birds, and fish was an inspiring source for the emerge of swarm intelligence [7]. Although swarm system consists of relatively unsophisticated individuals, they exhibit coordinated behavior that directs swarm towards the desired goal with no central component that manages the system as a whole.

Ant colony optimization (ACO) was founded on ant's ability to deploy a substance called pheromone for marking the discovered path between the food source and ant's nests. It is one of the oldest members of swarm intelligence family [8] but it is constantly being improved and applied to different problems [9–11].

Artificial bee colony (ABC) algorithm mimics the behavior of honey bee swarm. In this paradigm, three types of artificial bees perform search. Each type of bees has its particular role in the search process. ABC was originally proposed by Karaboga for problems of continuous optimization [12]. This algorithm proves to be robust and capable of solving high dimensionality problems [13–15].

Cuckoo search (CS) is an iterative approach that models search process by employing Levy flights (series of straight flight paths with sudden 90 degrees turn). It was first proposed by Yang and Deb [16] and proved to be a robust optimization technique [17], obtaining satisfying results in real-life optimizations like image thresholding [18].

Also, swarm intelligence metaheuristics which mimic the human search process were developed. Seeker optimization algorithm (SOA) is established on human memory, reasoning, past experience, and social interactions. It has been proven that the SOA is a robust technique for solving global numerical and real-life optimization problems [19] and is continuously being improved [20].

As a result of the literature survey, it can be concluded that for portfolio optimization problem there are some genetic algorithm (GA) implementations. However, there are only few swarm intelligence algorithms adapted for this problem. There are papers which refer to solving portfolio problem with nondominating sorting genetic algorithm (NSGA) which was first proposed by Srinivas and Deb [21]. Newer version NSGA-II improves the convergence and the spread of solutions in the population [22]. Lin et al. presented NSGA-II based algorithm with integer encoding for solving MV portfolio model with minimum transaction lots (MTL), fixed transaction costs (TC), and linear constraints on capital investments. The optimization of MV portfolio problem with cardinality and holding weights constraints by GA is shown in [23]. Soleimani et al. [24] presented GA with RAR crossover operator for solving MV portfolio problem where cardinality constraints, MTL, and constraints on sector capitalization are taken into account.

As mentioned, only few swarm intelligence metaheuristics exist for portfolio optimization. Deng and Lin presented ant colony optimization (ACO) for solving the cardinality constraints Markowitz MV portfolio model [25]. Haqiqi and Kazemi [26] employed the same algorithm to MV portfolio model. We emphasize on one ACO implementation based on the average entropy for real estate portfolio optimization [27]. This is one of the rare papers that incorporates entropy in portfolio model. Cura investigated PSO approach to cardinality constrained MV portfolio optimization [1]. The test data set contains weekly prices from March 1992 to September 1997 from the following five indexes: Hang Seng in Hong Kong, DAX 100 in Germany, FTSE 100 in UK, S&P 100 in USA, and Nikkei in Japan. The results of this study are compared with those from genetic algorithm, simulated annealing, and tabu search approaches and showed that PSO has potential in portfolio optimization. Zhu et al. [28] presented PSO algorithm for nonlinear constrained portfolio optimization with multiobjective functions. The model is tested on various restricted and unrestricted risky investment portfolios and a comparative study with GA is showed. PSO demonstrated high computational efficiency in constructing optimal risky portfolios and can be compared with other state-of-the-art algorithms.

ABC algorithm for mixed quadratic and integer programming problem of cardinality constrained MV portfolio model was presented by Wang et al. [29]. Some modifications of classical ABC algorithm for constrained optimization problems were adopted. The approach was tested on a standard benchmark data set and proved to be a robust portfolio optimizer.

One of the first implementations for portfolio optimization problem by the firefly algorithms was developed by Tuba et al. [30, 31]. Framework for solving this problem was devised. Metaheuristic was tested on standard five-asset data set. FA proved to be robust and effective technique for portfolio problem. Among other metaheuristics for portfolio problem, one approach based on neural networks (NN) should be distinguished [32].

In this paper, we propose a modified firefly algorithm (FA) for cardinality constrained mean-variance (CCMV) portfolio optimization with entropy constraint. FA was first introduced by Yang for unconstrained optimization [33]. Later, it was adapted [34] for solving various numerical and practical optimization problems [35–37].

We modified pure FA algorithm to adjust it for constrained problems and to improve its performance. We intensified exploration during early phase and eliminated it during late iterations when the proper part of the search space has been reached. Details will be given in Section 4.

The implementation of metaheuristics for the CCMV portfolio model with entropy constraint was not found in the literature survey. Thus, we conducted three experiments.

- First, we compared original FA with our modified mFA applied to portfolio optimization problem. We wanted to see what is the real improvement of our approach.
- Then, we compared results of our algorithm for the CCMV portfolio model with and without entropy constraint. In this test, we analyzed the influence of entropy constraint to portfolio diversification.
- Finally, in the third test, we made comparative analysis between our modified mFA and other state-of-the-art metaheuristics. We compared our proposed algorithm to Cura's PSO [1] and also to GA, TS, and SA, indirectly from [23].

The rest of the paper is organized as follows.  Section 2 presents mathematical formulations of variety portfolio optimization models. The presentation of the original FA is given in Section 3. Our proposed modified FA approach for the CCMV portfolio problem with entropy constraint is discussed in Section 4.  Section 5 first shows algorithm parameter settings that are used in experiments. Then, we present three experiments which we conducted along with the comparative analyses with other metaheuristics. Finally, Section 6 gives conclusions and recommendations for future research.

## 2. Portfolio Optimization Problem Definitions

Portfolio optimization, as one of the most important issues in modern financial management, tackles the problem of distribution of financial resources across a number of assets to maximize return and control the risk.

When making financial decisions, investors follow the principle of diversification by investing their capital into different types of assets. By investment in portfolios, rather than in single assets, the risk is mitigated by diversification of the investments, without negative impact on expected returns.

The essential form of portfolio optimization is formulated as bicriterion optimization problem where the reward, which is measured by the mean of return, should be maximized, while the risk, measured by the variance of return, should be minimized [38]. This problem deals with the selection of the portfolio of securities that minimizes the risk subject to the constraints, while guaranteeing a given level of returns [39].

By literature research many methods for solving portfolio problem can be found. Markowitz's standard mean-variance (MV) model choses one important objective function that is subject to optimization, while the remaining objective functions are being threated as constraints [40]. The key point in the MV formulation is to employ the expected returns of a portfolio as the investment return and the variance of returns of the portfolio as the investment risk [41]. Its basic assumptions are that the investors are rational with either multivariate normally distributed asset returns or in the case of arbitrary returns, a quadratic utility function [42]. This approach is widely adapted and plays an important role in the modern portfolio theory.

Markowitz's MV model considers the selection of risky portfolio as objective function, and the mean return of an asset as one of the constraints [43]. This model can mathematically be defined as

(5) $$\begin{array}{c} \min\sigma_{R_{p}}^{2}=\sigma_{p}^{2}=\overset{N}{\underset{i=1}{\sum }}\;\overset{N}{\underset{j=1}{\sum }}{\;\omega }_{i}\omega_{j}\text{Cov}\left(\bar{R_{i}}\;\bar{R_{j}}\right) \end{array}$$

subject to

(6) $$\begin{array}{c} \bar{R_{p}}=E\left(R_{p}\right)=\overset{N}{\underset{i=1}{\sum }}\omega_{i}\bar{R_{i}}\geq R, \end{array}$$

(7) $$\begin{array}{c} \overset{N}{\underset{i=1}{\sum }}\omega_{i}=1,\;\omega_{i}\geq 0,\;\;\forall i\in \left\{1,2,\ldots ,N\right\}, \end{array}$$

where *N* is the total number of available assets, $\bar{R_{i}}$ is the mean return on asset *i*, and $\text{Cov}(\bar{R_{i}}\;\bar{R_{j}})$ is covariance of returns of assets *i* and *j*, respectively. Constraint defined in (7) guarantees that the whole disposable capital is invested. Weight variable *ω*_*i*_ has a role of control parameter that determines the proportion of the capital that is placed in asset *i*. Weight variable has a real value in the range [0,1].

In the presented MV formulation, the objective is to minimize the portfolio risk *σ*_*p*_^2^, for a given value of portfolio expected return $\bar{R_{p}}$.

Efficient frontier model, which is often called single-objective function model, constructs only one evaluation function that models portfolio optimization problem. In this model, the desired mean return *R* is varying for the purpose of finding different objective function values. Risk aversion indicator *λ* ∈ [0,1] controls this process [28].

Efficient frontier definition is

(8) $$\begin{array}{c} \min\lambda \left[\overset{N}{\underset{i=1}{\sum }}\;‍\overset{N}{\underset{j=1}{\sum }}{\;\omega }_{i}\omega_{j}\text{Cov}\left(\bar{R_{i}}\;\bar{R_{j}}\right)\right]-\left(1-\lambda \right)\left[\overset{N}{\underset{i=1}{\sum }}\omega_{i}\bar{R_{i}}\right] \end{array}$$

subject to

(9) $$\begin{array}{c} \overset{N}{\underset{i=1}{\sum }}\omega_{i}=1\\ \omega_{i}\geq 0,\;\forall i\in \left\{1,2,\ldots ,N\right\}. \end{array}$$

In the presented formulation *λ* is critical parameter. It controls the relative importance of the mean return to the risk for the investor. When the value of *λ* is set to 0, mean return of portfolio is being maximized without considering the risk. Alternatively, when *λ* has a value of 1, risk of the portfolio is minimized regardless of the mean return. Thus, when the value of *λ* increases, the relative importance of the risk to the mean return for the investor rises, and vice versa.

Each *λ* value generates different objective function value which is composed of the mean return and the variance (risk). By tracing the mean return and variance intersections for different *λ*, a continuous curve can be drawn which is called an efficient frontier in the Markowitz's modern portfolio theory.

Another model worth mentioning is Sharpe ratio (SR) which uses the information from mean and variance of an asset [44]. In this model, the measure of mean return is adjusted with the risk and can be described by

(10) $$\begin{array}{c} \text{SR}=\frac{R_{p}-R_{f}}{\text{StdDev}\left(p\right)}, \end{array}$$

where *p* denotes portfolio, *R*_*p*_ is the mean return of the portfolio *p*, and *R*_*f*_ is a test available rate of return on a risk-free asset. StdDev(*p*) is a measure of the risk in portfolio (standard deviation of *R*_*p*_). By adjusting the portfolio weights *w*_*i*_, portfolio's Sharpe ratio can be maximized.

However, all three models: the MV, efficient frontier, and Sharpe ratio were constructed under strict and simplified assumptions that do not consider real-world parameters and limitations and as such are not always suitable for real applications. For these reason extended MV model is devised.

Extended MV formulation takes into account additional constraints such as budget, cardinality, transaction lots, and sector capitalization. These constraints model real-world legal and economic environment where the financial investments are being done [45]. Budget constraint controls the minimum and maximum total budget proportion that can be invested in particular asset. Cardinality constraint controls whether a particular asset will be included in the portfolio. The minimum transaction lots constraint ensures that each security can only be purchased in a certain number of units. Sector capitalization constraint directs the investments towards the assets that belong to the sectors where higher value of market capitalization can be accomplished. The review of this constraint is given in [24].

When all the above-mentioned constraints are being applied to the basic portfolio problem formulation, the problem becomes a combinatorial optimization problem whose feasible region is not continuous. Thus, the extended MV model belongs to the group of quadratic mixed-integer programming models. Its formulation is

(11) $$\begin{array}{c} \min\sigma_{R_{p}}^{2}=\sigma_{p}^{2}=\overset{N}{\underset{i=1}{\sum }}\;\overset{N}{\underset{j=1}{\sum }}{\;\omega }_{i}\omega_{j}\text{Cov}\left(\bar{R_{i}}\;\bar{R_{j}}\right), \end{array}$$

where

(12) ωi=xicizi∑j=1Nxjcjzj, i=1,…,N,

(13) ∑i=1Nzi=M≤N, M,N∈N,  zi∈{0,1}, i=1,…,N

subject to

(14) $$\begin{array}{c} \overset{N}{\underset{i=1}{\sum }}x_{i}c_{i}z_{i}\bar{R_{i}}\geq BR \end{array}$$

(15) $$\begin{array}{c} \overset{N}{\underset{i=1}{\sum }}x_{i}c_{i}z_{i}\leq B \end{array}$$

(16) $$\begin{array}{c} 0\leq B_{\text{lo}{\text{w}}_{i}}\leq x_{i}c_{i}\leq B_{\text{u}{\text{p}}_{i}}\leq B,\;i=1,\ldots ,N \end{array}$$

(17) ∑isWis≥∑is′Wis′∀ysys′≠0,  is,is′∈{1,2,…,N},   s,s′∈{1,2,…,S},  s<s′,

where

(18) $$\begin{array}{c} y_{s}=\{\begin{array}{cc} 1 & \text{if}\;\;\underset{i_{s}}{\sum }z_{i}>0\\ 0 & \text{if}\;\;\underset{i_{s}}{\sum }z_{i}=0, \end{array} \end{array}$$

In (11)–(18) *M* represents the number of selected assets among possible *N* assets. *B* is the total disposable budget, and *B*_low_*i*__ and *B*_up_*i*__ are lower and upper limits of the budget that can be invested in asset *i*, respectively. *S* represents the number of sectors in the market where the investment is being placed, *c*_*i*_ is the size of transaction lot for asset *i*, and *x*_*i*_ denotes the number of such lots (of asset *i*) that is purchased.

Decision variable *z*_*i*_ is used to apply cardinality constraint: *z*_*i*_ is equal to 1 if an asset *i* is present in the portfolio, otherwise its value is 0. Equation (17) models sector capitalization constraint. Despite the fact that a certain sector has high capitalization, security from this sector that has low return and/or high risk must be excluded from the final portfolio's structure. To make such exclusion, variable *y*_*s*_ is defined and it has a value of 1 if the corresponding sector has at least one selected asset, and 0 otherwise. In (17) *i*_*s*_ is a set of assets which can be found in sector *s*. Sectors are sorted in descending order by their capitalization value.

Entropy was introduced by Jaynes [46] for wide application in optimization, crystallography in the beginning, networks [47], and so forth, but it also becomes an important tool in portfolio optimization and asset pricing. Entropy is widely recognized measure of portfolio diversification [2]. In multiobjective portfolio optimization models, the entropy can be used as an objective function. Here, we will address entropy as diversity constraint in portfolio models, because we employed it in portfolio model that is used for testing of our modified FA approach.

The entropy constraint defines lower bound *L*_*E*_ of entropy *E*(*P*) of portfolio *P* according to the following equation [48]:

(19) $$\begin{array}{c} E\left(P\right)=-\overset{N}{\underset{i=1}{\sum }}x_{i}\ln x_{i}\geq L_{E}, \end{array}$$

where *N* is the number of assets in portfolio *P* and *x*_*i*_ is weight variable of the security *i*.

In one extreme, when the weigh variable of only one asset in portfolio *P* is 1, and for the rest of the assets is 0, *E*(*P*) reaches its minimum at −1∗ln⁡1 = 0 [49]. This is the least diverse scenario. Contrarily, in the most diverse condition that, for ∀*i*,  *x*_*i*_ = 1/*N*, *E*(*P*) obtains its maximum in −*N*(1/*N*ln⁡1/*N*) = ln⁡*N*. According to this, *L*_*E*_ is in the range [0, ln⁡*N*]. Greater value of entropy denotes better portfolio's diversity, and *L*_*E*_ is used to make sure that the diversity of *P* is not too low.

Entropy constraint equation (19) can be transformed into the upper-bound constraint [49]:

(20) $$\begin{array}{c} F\left(P\right)=e^{-E(P)}=e^{\sum_{i=1}^{N}x_{i}\ln x_{i}}\leq U_{E}. \end{array}$$

As shown previously, 0 ≤ *E*(*P*) ≤ ln⁡*N*, which implicates that 0 ≥ −*E*(*P*)≥−ln⁡*N*. Then, the condition *e* ^0^ = 1 ≥ *e* ^−*E*(*P*)^ = *F*(*P*) ≥ *e* ^−ln⁡*N*^ = 1/*N* holds. Thus, the range of upper-bound constraint *U*_*E*_ is [1/*N*, 1].

In this paper, for testing purposes, we used model which employs some of the constraints that can be found in the extended MV formulation. In the experimental study, we implemented modified FA for optimizing cardinality constrained mean-variance model (CCMV) which is derived from the standard Markowitz's and the efficiency frontier models.

We were inspired by Usta's and Kantar's multiobjective approach based on a mean-variance-skewness-entropy portfolio selection model (MVSEM) that employs Shannon's entropy measure to the mean-variance-skewness portfolio model (MVSM) to generate a well-diversified portfolio [50, 51]. Thus, we added entropy measure to the CCMV portfolio formulation to generate well-diversified portfolio.

Formulation of the CCMV model with entropy constraint is

(21) $$\begin{array}{c} \min\lambda \left[\overset{N}{\underset{i=1}{\sum }}\;\overset{N}{\underset{j=1}{\sum }}{\;x}_{i}x_{j}\sigma_{i,j}\right]-\left(1-\lambda \right)\left[\overset{N}{\underset{i=1}{\sum }}x_{i}\mu_{i}\right] \end{array}$$

subject to

(22) $$\begin{array}{c} \overset{N}{\underset{i=1}{\sum }}x_{i}=1, \end{array}$$

(23) $$\begin{array}{c} \overset{N}{\underset{i=1}{\sum }}z_{i}=K, \end{array}$$

(24) $$\begin{array}{c} {ɛ}_{i}z_{i}\leq x_{i}\leq \delta_{i}z_{i},\;z\in \left\{0,1\right\},\;i=1,2,3,\ldots ,N, \end{array}$$

(25) $$\begin{array}{c} -\overset{N}{\underset{i=1}{\sum }}z_{i}x_{i}\ln x_{i}\geq L_{E}. \end{array}$$

As already mentioned in this section, *N* is the number of potential securities that will be included in portfolio, *λ* is risk aversion parameter, *x*_*i*_ and *x*_*j*_ are weight variables of assets *i* and *j*, respectively, *δ*_*i*,*j*_ is their covariance, and *μ*_*i*_ is *i*th asset's return. *K* is the desired number of assets that will be included in the portfolio. Decision variable *z*_*i*_ controls whether the asset *i* will be included in portfolio. If its value is 1, asset *i* is included, and if the value is 0, asset *i* is excluded from the portfolio. *ɛ* and *δ* are lower and upper bounds of the asset that is included in portfolio and they make sure that the asset's proportion in the portfolio is within the predefined range.

We applied entropy constraint equation (25) with lower bounds, as in (19), to ensure that the diversity of portfolio is not too low. *L*_*E*_ is lower bound of the entropy in the range [0, ln⁡*K*]. In (25), *z*_*i*_ ensures that only assets that are included in portfolio are taken into account.

From the CCMV formulation with entropy constraint it can be seen that this problem belongs to the group of mixed quadratic and integer programming problems. It employs both real and integer variables with equity and inequity constraints.

## 3. Presentation of the Original FA

Firefly algorithm (FA) was originally proposed by Yang in 2008 [33], with later improvements [52]. It was applied to continuous [53], discrete [54], and mixed [55] optimization problems. The emergence of this metaheuristic was inspired by the social and flashing behavior of fireflies.

Fireflies inhabit moderate and tropical climate environments all around the word. Their flashing behavior has many different roles. Synchronized flashing by males is unique in the animal world and involves a capacity for visually coordinated, rhythmically coincident, and interindividual behavior. Also, flashing is used to alleviate communication for mating and to frighten the predators. These flashing properties can be incorporated into swarm intelligence metaheuristic in such a way that they are associated with the objective function which is subject to optimization.

With respect to the facts that the real firefly system is sophisticated and that the metaheuristics are approximations of real systems, three idealized rules are applied with the goal to enable algorithm's implementation [33]: (1) all fireflies are unisex, so the attractions between fireflies do not depend on their sex; (2) attractiveness of a firefly is directly proportional to their brightness, and the less brighter firefly will move towards the brighter one. Brightness increases as the distance between fireflies decreases; (3) the brightness of a firefly is determined by the value of objective function. For minimization problems, brightness increases as the objective function value decreases. There are also other forms of brightness which can be defined similar to fitness function in genetic algorithms (GA) [6].

In the implementation of FA, one of the most important issues that should be considered is the formulation of attractiveness. For the sake of simplicity, a good approximation is that the attractiveness of a firefly is determined by its brightness which depends on the encoded objective function.

In the case of maximization problems, the brightness of a firefly at a particular location *x* can be chosen as *I*(*x*) ~ *f*(*x*), where *I*(*x*) is the attractiveness and *f*(*x*) is the value of objective function at this location. Otherwise, if the goal is to minimize function, the following expression can be used:

(26) $$\begin{array}{c} I\left(x\right)=\{\begin{array}{cc} \frac{1}{f\left(x\right)}, & \text{if}\;f\left(x\right)>0\\ 1+\left|f\left(x\right)\right|, & \text{otherwise}. \end{array} \end{array}$$

The variations of light intensity and attractiveness are monotonically decreasing functions because as the light intensity and the attractiveness decrease, the distance from the source increases, and vice versa. This can be formulated as [56]

(27) $$\begin{array}{c} I\left(r\right)=\frac{I_{0}}{1+\gamma r^{2}}, \end{array}$$

where *I*(*r*) is the light intensity, *r* is distance, and *I*_0_ is the light intensity at the source. Besides that, the air also absorbs part of the light, and the light becomes weaker. Air absorption is modeled by the light absorption coefficient *γ*.

In most FA implementations that can be found in the literature survey, the combined effect of both the inverse square law and absorption can be approximated using the following Gaussian form:

(28) $$\begin{array}{c} I\left(r\right)=I_{0}e^{-\gamma r^{2}}. \end{array}$$

Attractiveness *β* of a firefly is relative because it depends on the distance between the firefly and the beholder. Thus, it varies with the distance *r*_*i*,*j*_ between fireflies *i* and *j*. The attractiveness is direct proportional to fireflies light intensity (brightness), as shown in the following:

(29) $$\begin{array}{c} \beta \left(r\right)=\beta_{0}e^{-\gamma r^{2}}, \end{array}$$

where *β*_0_ is the attractiveness at *r* = 0. Equation (29) determines a characteristic distance $\Gamma =1/\sqrt{\gamma }$ over which the attractiveness changes significantly from *β*_0_ to *β*_0_ *e* ^−1^.

But, in practical applications, the above expression is usually replaced with

(30) $$\begin{array}{c} \beta \left(r\right)=\frac{\beta_{0}}{1+\gamma r^{2}}. \end{array}$$

Main reason for this replacement is that the calculation of exponential function in (29) demands much more computational power than simple division in (30).

The movement of a firefly *i* (its new position in iteration *t* + 1) towards the brighter, and thus more attractive firefly *j* is calculated using

(31) $$\begin{array}{c} x_{i}\left(t+1\right)=x_{i}\left(t\right)+\beta_{0}r^{-\gamma r_{i,j}^{2}}\left(x_{j}-x_{i}\right)+\alpha \left(\kappa -0.5\right), \end{array}$$

where *β*_0_ is attractiveness at *r* = 0, *α* is randomization parameter, *κ* is random number drawn from uniform or Gaussian distribution, and *r*_*i*,*j*_ is distance between fireflies *i* and *j*. The positions of fireflies are updated sequentially by comparing and updating each pair of them at every iteration.

The distance between fireflies *i* and *j* is calculated using Cartesian distance form [56]:

(32) $$\begin{array}{c} r_{i,j}=||x_{i}-x_{j}||=\sqrt{\overset{D}{\underset{k=1}{\sum }}\left(x_{i,k}-x_{j,k}\right)}, \end{array}$$

where *D* is the number of problem parameters. For most problems, *β*_0_ = 0 and *α* ∈ [0,1] are adequate settings.

The parameter *γ* has crucial impact on the convergence speed of the algorithm. This parameter shows the variation of attractiveness and in theory it has a value of [0, +*∞*), but in practice it is determined by the characteristic distance Γ of the system that is being optimized. In most implementations *γ* parameter varies between 0.01 and 100.

There are two special cases of the FA, and they are both associated with the value of *γ* as follows [33]:

- if *γ* = 0, then *β* = *β*_0_. That means that the air around firefly is completely clear. In this case, *β* is always the largest it could possibly be, and fireflies advance towards other fireflies with the largest possible steps. The exploration-exploitation is out of balance because the exploitation is maximal, while the exploration is minimal;
- if *γ* = *∞*, then *β* = 0. In this case, there is a thick fog around fireflies and they could not see each other. The movement is performed in a random steps, and exploration is more intensive with practically no exploitation at all.

The pseudocode for the original FA is given as Algorithm 1.

In the presented pseudocode, *SN* is total number of fireflies in the population, *IN* is total number of algorithm's iterations, and *t* is the current iteration.

## 4. Proposed mFA for the CCMV Portfolio Problem with Entropy Constraint

As mentioned in Section 1, we propose a modified firefly algorithm for cardinality constrained mean-variance portfolio optimization with entropy constraint.

By analyzing FA we noticed that, as most other swarm intelligence algorithms, the pure version of the algorithm, developed for unconstrained problems, exhibits some deficiencies when applied to constrained problems. In the early cycles of algorithm's execution established balance between exploitation and exploration is not completely appropriate for this class of problems. During early phase exploration is not intensive enough. However, during late cycles when FA was able to discover the right part of the search space, the exploration is no longer needed. To control whether the exploration will be triggered or not, we introduced exploration breakpoint *EBP* control parameter.

In this section, we show implementation details of our modified FA algorithm which we named mFA.

### 4.1. Initialization Phase and Fitness Calculation

At the initialization step, FA generates random population of *SN* fireflies (artificial agents) using

(33) $$\begin{array}{c} x_{i,j}=lb_{j}+\text{rand}\left(0,1\right)*\left(ub_{j}-lb_{j}\right), \end{array}$$

where *x*_*i*,*j*_ is the weight of the *j*th portfolio's asset of the *i*th agent, rand(0,1) is random number uniformly distributed between 0 and 1, and *ub*_*j*_ and *lb*_*j*_ are upper and lower weight bounds of the *j*th asset, respectively.

If the initially generated value for the *j*th parameter of the *i*th firefly does not fit in the scope [*lb*_*j*_, *ub*_*j*_], it is being modified using the following expression:

(34) $$\begin{array}{c} \text{if}\;\left(x_{i,j}\right)>ub_{j},\;\text{then}\;x_{i,j}=ub_{j}\\ \text{if}\;\left(x_{i,j}\right)<lb_{j},\;\text{then}\;x_{i,j}=lb_{j}. \end{array}$$

Moreover, in the initialization phase, decision variables *z*_*i*,*j*_  (*i* = 1,…, *SN*, *j* = 1,…, *N*) are also initialized for each firefly agent *i*. *N* is the number of potential assets in portfolio. According to this, each firefly is modeled using 2∗*N* dimensions. *z*_*i*_ is a binary vector, with values 1, when an asset is included in portfolio, and 0, when it is excluded from it.

Decision variables are generated randomly by applying

(35) $$\begin{array}{c} z_{i,j}=\{\begin{array}{cc} 1, & \text{if}\;\phi <0.5\\ 0 & \text{if}\;\phi \geq 0.5, \end{array} \end{array}$$

where *ϕ* is random real number between 0 and 1.

To guarantee the feasibility of solutions, we used similar arrangement algorithm as proposed in [1]. The arrangement algorithm is applied first time in the initialization phase.

In the arrangement algorithm, *i* is the current solution that consists of *Q* the distinct set of *K*_i_* assets in the *i*th solution, *z*_*i*,*j*_ is the decision variable of asset *j*, and *x*_*i*,*j*_ is the weight proportion for asset *j*. Arrangement algorithm pseudocode is shown as Algorithm 2.

For the constraint ∑_*i*=1_^*N*^ *x*_*i*_ = 1 we set *ψ* = ∑_*j*∈*Q*_ *x*_*i*,*j*_ and put *x*_*i*,*j*_ = *x*_*i*,*j*_/*ψ* for all assets that satisfy *j* ∈ *Q*. The same approach for satisfying this constraint was used in [1]. To make sure that each asset's proportion is within predefined lower and upper bounds, *ɛ* and *δ*, respectively, we used *if* *x*_*i*,*j*_ > *δ*_*i*,*j*_ *then x*_*i*,*j*_ = *δi*, *j* and *if* *x*_*i*,*j*_ < *ɛ*_*i*,*j*_ *then x*_*i*,*j*_ = *ɛ*_*i*,*j*_.

We did not apply *c*-value based approach for adding and removing assets from the portfolio as in [1]. According to our experiments, using *c*-value does not improve FA performance. It only increases computational complexity.

In modified FA, the fitness is employed to model the attractiveness of the fireflies. Attractiveness is directly proportional to the fitness.

After generating *SN* number of agents, fitness value is calculated for each firefly in the population. Fitness (brightness) is calculated as in the original FA implementation (26).

In the initialization phase, for each firefly in the population, constraint violation CV is being calculated. CV is a measure of how much the agents violate constraints in the problem definition:

(36) $$\begin{array}{c} \text{C}{\text{V}}_{i}=\underset{g_{j}(x_{i})>0}{\sum }g_{j}\left(x_{i}\right)+\overset{m}{\underset{j=q+1}{\sum }}h_{j}\left(x_{i}\right). \end{array}$$

CV calculation is necessary, because it is later used for performing selection based on Deb's method [57, 58].

### 4.2. Firefly Movement

The movement of a firefly *i* towards the firefly that has a higher fitness *j* is calculated as in the original FA implementation [56]:

(37) $$\begin{array}{c} x_{i}\left(t+1\right)=x_{i}\left(t\right)+\beta_{0}r^{-\gamma r_{i,j}^{2}}\left(x_{j}-x_{i}\right)+\alpha \left(\kappa -0.5\right), \end{array}$$

where *x*_*i*_(*t* + 1) is new solution generated in iteration (*t* + 1), *β*_0_ is attractiveness at *r* = 0, *α* is randomization parameter, *κ* is random number drawn from uniform or Gaussian distribution, and *r*_*i*,*j*_ is distance between fireflies *i* and *j*.

Also, when moving a firefly, new decision variables are calculated:

(38) $$\begin{array}{c} z_{i,k}^{t+1}=\text{round}\left(\frac{1}{1+e^{-z_{i,k}^{t}+\phi_{i,j}(z_{i,k}^{t}-z_{j,k}^{t})}}-0.06\right), \end{array}$$

where *z*_*i*,*k*_^*t*+1^ is decision variable for the *k*th asset of the new solution, *z*_*i*,*k*_ is a decision variable of the *k*th parameter of the old solution, and *z*_*j*,*k*_ is decision variable of *k*th parameter of the brighter firefly *j*.

It should be noticed that the decision variables in the employed bee phase are generated differently than in the initialization phase equation (35).

After the new *i*th solution is generated in exploitation process using (37) and (38) the winner between new *x*_*i*_(*t* + 1) and old *x*_*i*_(*t*) solution is retained using the selection process based on Deb's rules.

### 4.3. Exploration

As mentioned before, we noticed insufficient exploration power in the original FA implementation, particularly in early iterations of algorithm's execution. In this phase of algorithm's execution, exploitation-exploration balance is not well established for this type of problems. This balance was also discussed in [14]. Thus, we adopted mechanism similar to scout bee with *limit* parameter from the ABC metaheuristic.

We introduced parameter abandonment threshold (*AT*) that represents the allowed predetermined number of unsuccessful tries to improve particular solution. When a potential solution (firefly) is stagnating (not being improved) for *AT* iterations, it is replaced by a new, random one using (33), (34), and (35). Hence, fireflies that exploited exhausted solutions are transformed into scouts that perform the exploration process. The value of *AT* is empirically determined and will be shown in the experimental section.

Also, during late iterations, with the assumption that the right part of the search space has been found, the intensive exploration is not needed any more. In that case, the exploration is not being triggered. For this purpose, we introduce new control parameter, exploration breakpoint (*EBP*) which controls whether the exploration will be triggered. The discussion of this parameter is also given in experimental section.

Also, we should note that the parameter *α* for FA search process is being gradually decreased from its initial value according to

(39) $$\begin{array}{c} \alpha \left(t\right)=\left(1-\left(1-\left({\left(\frac{10^{-4}}{9}\right)}^{1/IN}\right)\right)\right)*\alpha \left(t-1\right), \end{array}$$

where *t* is the current iteration and *IN* is the maximum number of iterations.

Pseudocode of mFA is given as Algorithm 3. Some implementation's details are omitted for the sake of simplicity.

In the pseudocode, *SN* is total number of fireflies in the population, *IN* is total number of algorithm's iterations, and *t* is the current iteration. As explained, *AT* is the maximum number of unsuccessful attempts to improve particular solution after which it will be considered exhausted and replaced by a new, random solution.

## 5. Algorithm Settings and Experimental Results

In this section, we first present parameter settings which were adjusted for testing purposes of our proposed mFA. Then, we show experimental results, discussion, and comparative analysis with other state-of-the-art algorithms.

### 5.1. Parameter Settings

To test the performance and robustness of our modified FA, we set algorithm parameters similar to [1]. Number of firefly agents in the population *SN* is calculated by employing the following expression:

(40) $$\begin{array}{c} SN=20\sqrt{N}, \end{array}$$

where *N* is the number of potential assets in portfolio.

The value of maximum number of iterations *IN* in one algorithm's run is set according to

(41) $$\begin{array}{c} IN=\frac{1000*N}{SN}. \end{array}$$

As mentioned in Section 4, to improve the exploration power of the original FA, we introduced parameter *AT* with corresponding counters *UIC*_*i*_  (*i* = 1,2,…, *SN*) that count how many times a particular firefly agent unsuccessfully tried improvement. When the value of *UIC*_*i*_ reaches predetermined abandonment threshold *AT*, corresponding agent is being replaced by a random agent. *AT* is determined by the values of *SN* and *IN*, like in [14]:

(42) $$\begin{array}{c} AT=\frac{IN}{SN}=\frac{\left(1000*N\right)/SN}{20\sqrt{N}}. \end{array}$$

Exploration breakpoint *EBP* controls whether or not the exploration will be triggered. According to our experimental tests, modified FA generates worse results if the exploration is triggered during late iterations. In most of the runs, the algorithm is able to find a proper part of the search space during early cycles, and exploration during late cycles is not useful. To the contrary, it just relaxes the exploitation. *EBP* is empirically set to *IN*/2.

FA search process parameter *α* is set to start at 0.5, but it is being gradually decreased from its initial value according to (39).

The promising approaches for handling equality constraints include dynamic, self-adaptive tolerance adjustment [59]. When this tolerance is included, the exploration is enhanced by exploring a larger space.

In modified FA implementation, besides the adoption of arrangement algorithm we used (4) and violation limit *υ* for handling constraints. Good experimental results are obtained by starting with a relatively large *υ* value, which is gradually decreasing through the algorithm's iterations. It is very important to chose the right value for *υ*. If the chosen value is too small, the algorithm may not find feasible solutions, and otherwise the results may be far from the feasible region [14].

We used the following dynamic settings for the *υ*:

(43) $$\begin{array}{c} \nu \left(t+1\right)=\frac{\nu \left(t\right)}{dec}, \end{array}$$

where *t* is the current generation and *dec* is a value slightly larger than 1. For handling equality constraints, we set initial value for *υ* to 1.0, *dec* to 1.001 and the threshold for *υ* to 0.0001 like in [3].

For generating heuristics efficient frontier, we used *ξ* = 51 different *λ* values. Thus, we set Δ*λ* to 0.02 because *λ* in the first algorithm's run is 0 and in the last is 1.

We also set number of assets that will be included in portfolio *K* to 10, lower asset's weight *ɛ* to 0.01, and upper asset's weight *δ* to 1.

Since the entropy lower bound depends on the number of assets that will be included in portfolio, we set *L*_*E*_ in the range of [0, ln⁡10].

We present again short modified FA pseudocode as Algorithm 4, but this time with the emphasis on the parameter settings.

For making better distinction between parameters, we divided algorithm parameters into four groups: modified FA global control parameters, FA search parameters, portfolio parameters, and constraint-handling parameters.

Parameters are summarized in Table 1.

### 5.2. Experimental Results and Comparative Analysis

In this subsection, we show the results obtained when searching the general efficient frontier that provides the solution for the problem formulated in (21)–(25). The test data were downloaded from http://people.brunel.ac.uk/~mastjjb/jeb/orlib/portinfo.html.

Benchmark data refers to the weekly stock prices from March 1992 to September 1997 for the indexes: the Hong Kong Hang Seng with 31 assets, the German Dax 100 with 85 assets, the British FTSE 100 with 89 assets, the US S&P 100 with 98 assets, and the Japanese Nikkei with 225 assets.

We adapted test data and stored it in Excel spreadsheets. For all indexes, we used the following data: mean return, standard deviation of return for each asset, and correlation for each possible pair of assets. Also, for generating standard efficiency frontier, we used mean return and variance of return for each security.

Since *SN*, *MCN*, and *AT* parameters depend on the problem size *N* (number of securities in the test), we show exact values used for all indexes (tests) in Table 2. Formula results are rounded to the closest integer values.

Lower bound for entropy for all benchmarks set is in the range between 0 and ln⁡10, because *K* is set to 10 for all test cases.

We conducted tests on Intel CoreTM i7-4770 K processor @4 GHz with 16 GB of RAM memory, Windows 7 x64 Ultimate 64 operating system and Visual Studio 2012 with NET 4.5 Framework.

When sets of Pareto optimal portfolios obtained with modified FA are taken, heuristic efficient frontier can be traced. In this paper, we compare the standard efficient frontiers for five real-world benchmark sets mentioned above with the heuristic efficient frontier for the same data set. For comparison of standard and heuristic efficiency frontier, we use mean Euclidean distance, variance of return error, and mean return error as in [1]. We also give the execution time of modified FA for each benchmark on our computer system platform.

For calculation purposes of mean Euclidean distance, let the pair (*v*_*i*_^*s*^, *r*_*i*_^*s*^) = (*i* = 1,2, 3,…, 2000) denote the variance and mean return of the point in the standard efficient frontier, and the pair (*v*_*j*_^*h*^, *r*_*j*_^*h*^) = (*j* = 1,2, 3,…, *ξ*) represents the variance and mean return of the point in the heuristic efficient frontier. Then, the index *i*_*j*_ of the closest standard efficiency frontier point to the heuristic efficiency frontier point, denoted as (*v*_*i*,*j*_^*s*^, *r*_*i*,*j*_^*s*^), is calculated using Euclidean distance by

(44) ij=arg min⁡i=1,2,…,2000⁡((vis−vjh)2+(ris−rjh)2)             j=1,2,3,…,ξ.

Using (44), mean Euclidean distance is defined as

(45) $$\begin{array}{c} \frac{\sum_{j=1}^{\xi }\sqrt{{\left(v_{i,j}^{s}-v_{j}^{h}\right)}^{2}+{\left(r_{i,j}^{s}-r_{j}^{h}\right)}^{2}}}{\xi }. \end{array}$$

In addition to mean Euclidean distance, we used two other measures to test modified FA, variance of return error and mean return error.

Variance of return error is defined as

(46) $$\begin{array}{c} \left(\overset{\xi }{\underset{j=1}{\sum }}\frac{\left|v_{i,j}^{s}-v_{j}^{h}\right|}{v_{j}^{h}}\right)\frac{1}{\xi }. \end{array}$$

Mean return error is calculated as

(47) $$\begin{array}{c} \left(\overset{\xi }{\underset{j=1}{\sum }}\frac{\left|r_{i,j}^{s}-r_{j}^{h}\right|}{r_{j}^{h}}\right)\frac{1}{\xi }. \end{array}$$

For testing purposes, we conducted three experiments. In the first experiment, we compared mFA with the original FA for CCMV problem with entropy constraint. Second experiment refers to comparative analysis between mFA for CCMV problem with and without entropy constraint. Finally, in the third experiment, we perform comparative analysis between our modified mFA and other state-of-the-art metaheuristics. We compared our proposed algorithm to Cura's PSO [1] and also to GA, TS, and SA, indirectly from [23].

We first wanted to analyze how our mFA compares to the original FA when optimizing CCMV portfolio model with entropy constraint. Thus, we also implemented original FA for this purpose. We compared mean Euclidean distance, variance of return error, and mean return error. These performance indicators were described above. We also calculated computational time for both algorithms. This time is comparable since the same computer platform was used for testing both original FA and mFA. This comparison is shown in Table 3. For better distinction between indicator values, we marked better results in bold.

As can be seen from Table 3, mFA obtains better results for almost all benchmarks. Only for variance of return error and mean return error indicators for *FTSE*100 index test, original FA managed to achieve better values. For this benchmark, exploration in early iterations is unnecessary because the algorithm quickly converges to the right part of the search space, and the firefly agents are being wasted on exploration.

All three indicators, mean Euclidean distance, variance of return error, and mean return error, are significantly better for mFA tests for *HangSeng*, DAX100, S&P100, and *Nikkei* indexes. Since mFA utilizes exploration at early cycles, computation time for all tests is worse (higher) than for the original FA implementation.

In the second experiment, we compared our mFA for CCMV problem with and without entropy constraint to show how the entropy constraint influences the results. CCMV formulation without entropy constraint is defined in (21)–(24).

According to the results presented in Table 4, it is clear that the entropy constraint slightly effects the portfolio's performance. In the CCMV optimization with entropy constraint, for *HangSeng* and S&P tests, mean Euclidean distance is slightly better, so the portfolio is better diversified. For other three tests, the results obtained for this indicator are the same. Also, for *HangSeng*, DAX100, *FTSE*100, and S&P100 indexes, optimization of the model with entropy gains better variance of return error and mean return error values. Only for *Nikkei* tests, those indicators have better value for CCMV model optimization without entropy constraint. Since the algorithm takes extra time to calculate the entropy constraint, execution time for CCMV with entropy is higher for all tests except *HangSeng* because this benchmark incorporates less securities than the other benchmarks.

The implementation of metaheuristics for CCMV portfolio model with entropy constrained could not be found in the literature. Thus, in the third experiment, we compared our mFA approach with metaheuristics for CCMV portfolio formulation which did not employ entropy. This model is defined by (21)–(24). We note that this test is not objective indicator of mFA's effectiveness compared to the other algorithms.

We compared mFA with tabu search (TS), genetic algorithm (GA), simulated annealing (SA), from [23], and PSO from [1] for the same set of benchmark data. As in the first two experiments, for performance indicators, we used mean Euclidean distance, variance of return error, and mean return error. Parameter settings for our mFA are given in Tables 1 and 2 and are comparable to parameters for other four compared algorithms that can be found in [1, 23]. We also give computational time for mFA, but those results are incomparable with results for other metaheuristics because we used different computer platform and portfolio model. In experiments in [1], Pentium M 2.13 GHz computer with 1 GB RAM was used. In the results table, best obtained results of all five heuristics are printed bold.

Other metaheuristic implementations for CCMV portfolio problem, such as modified ABC [29] and hybrid ABC (HABC) [41] that have similar performance, can be found in the literature.

If we consider that the optimization of CCMV with entropy constraint obtains only slightly better results than optimization of CCMV model without entropy in Table 4, the experimental results in Table 5 could be used for comparison of the performance of mFA with other metaheuristics in some sense.

The experimental results presented in Table 5 prove that none of the four algorithms which we used for comparisons has distinct advantages but that on average, mFA is better approach than other four metaheuristics.

mFA obtains better (smaller) mean Euclidean distance for all five benchmark sets. In *HangSeng* and *FTSE*100 benchmarks, mFA is better than all four algorithms for all three indicators, mean Euclidean distance, variance of return error, and mean return error. For those benchmarks, mFA was able to approximate the standard efficient frontier with the smallest mean return and variance of return error, and under the same risk values.

Second best algorithm shown in Table 5 is GA which obtains best mean return error and variance of return error in DAX100 and S&P100 tests, respectively. TS shows best performance for mean return error indicator in S&P100 benchmark, SA for mean return error in *Nikkei* test, while PSO proves to be most robust for variance of return error in DAX100 index.

From the presented analysis it can be concluded that our approach obtained results for CCMV portfolio optimization problem that can be more valuable for the investors: mFA's results are more accurate and the generated investment strategy is able to more efficiently diversify the risk of the portfolio.

## 6. Conclusions

In this paper we presented modified firefly algorithm (mFA) for cardinality constrained mean-variance portfolio optimization problem with entropy constraint. We adopted from the ABC algorithm *limit* parameter that controls and directs the exploration process. Original firefly algorithm suffers from low exploration power at early iterations of algorithm's execution for this type of problems. By introducing exploration into this phase of execution, we overcome this deficiency. However, during late cycles when the right part of the search space was reached, the exploration is no longer needed. To control whether the exploration will be triggered or not, we introduced exploration breakpoint *EBP* control parameter.

Since swarm intelligence implementations for the CCMV portfolio model with entropy constraint could not be found in the literature, we conducted three experiments. In the first experiment, to measure the enhancement gained by our modifications, we compared our proposed mFA with the original FA for CCMV model. Test results show that our modifications completely rectified original FA deficiencies. To show how the entropy constraint affects the CCMV portfolio model, in the second experiment we compared results of the mFA for CCMV models with and without entropy constraints. Test results proved that inclusion of the entropy constraint is justified since it ensures portfolio diversification and, consequently, quality of results enhancement. Finally, to test the performance and robustness of our algorithm, we compared it with four other state-of-the-art algorithms from [1] (and indirectly [23]). Our proposed algorithm proved almost uniformly better compared to genetic algorithm, tabu search, simulated annealing, and particle swarm optimization. This all establishes modified firefly algorithm as a usable tool for cardinality constrained mean-variance portfolio optimization problem with entropy constraint.

Future research may include application of the proposed mFA to other portfolio optimization models and formulations with different constraints. Also, additional modifications of the FA algorithm can be investigated for possible further improvement of results.

## Figures and Tables

**Algorithm 1:**
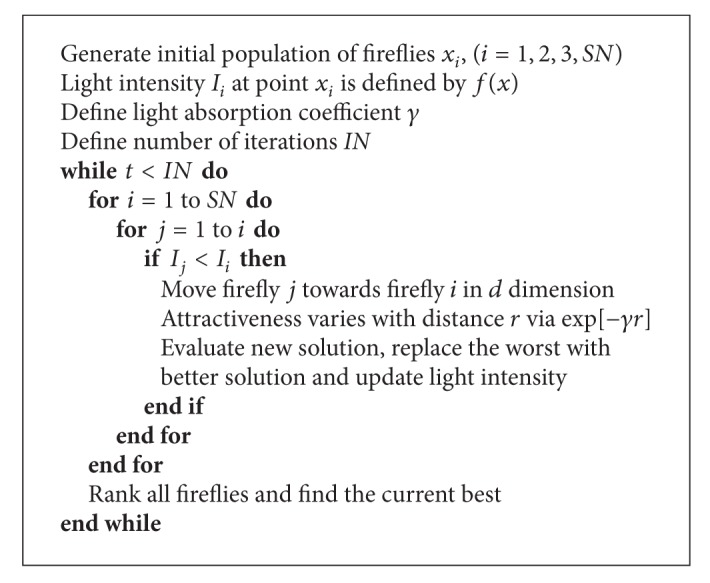
Original firefly algorithm.

**Algorithm 2:**
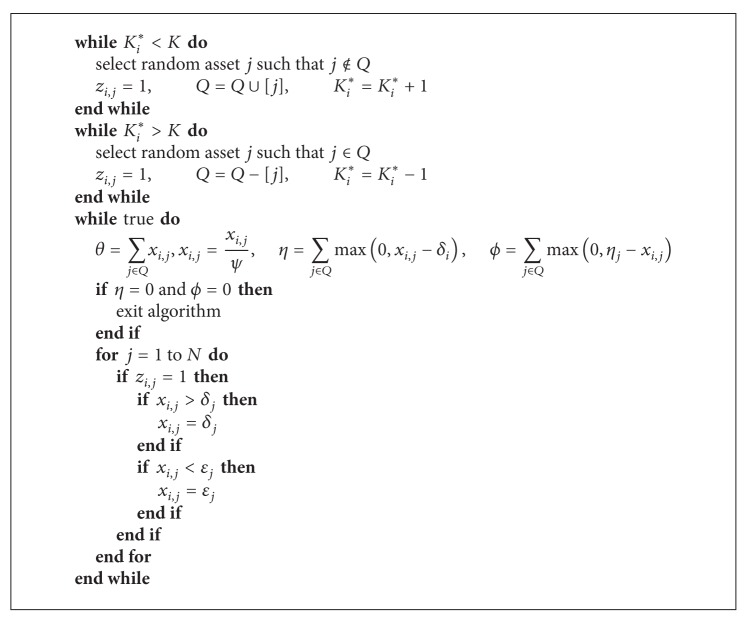
Arrangement algorithm.

**Algorithm 3:**
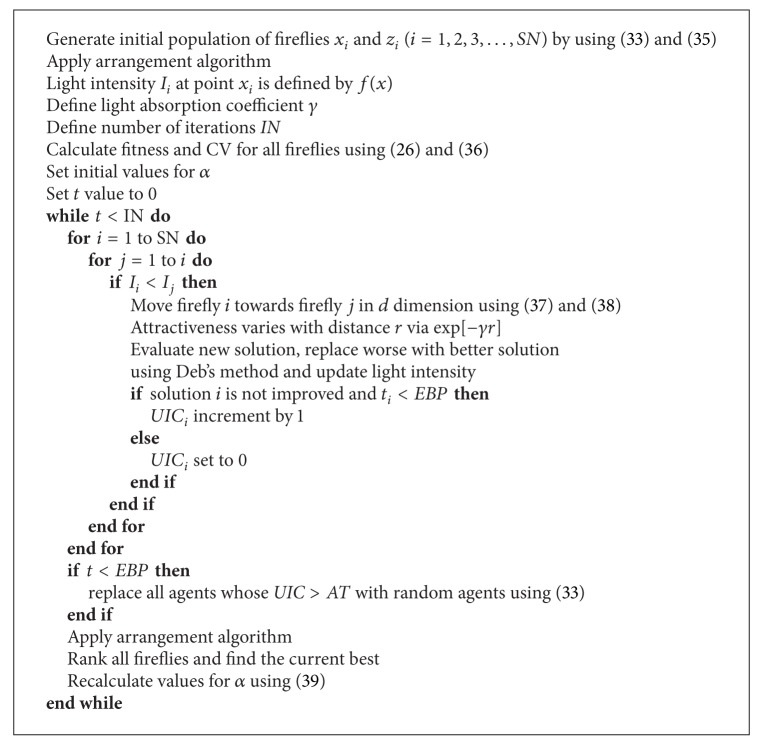
Modified firefly algorithm.

**Algorithm 4:**
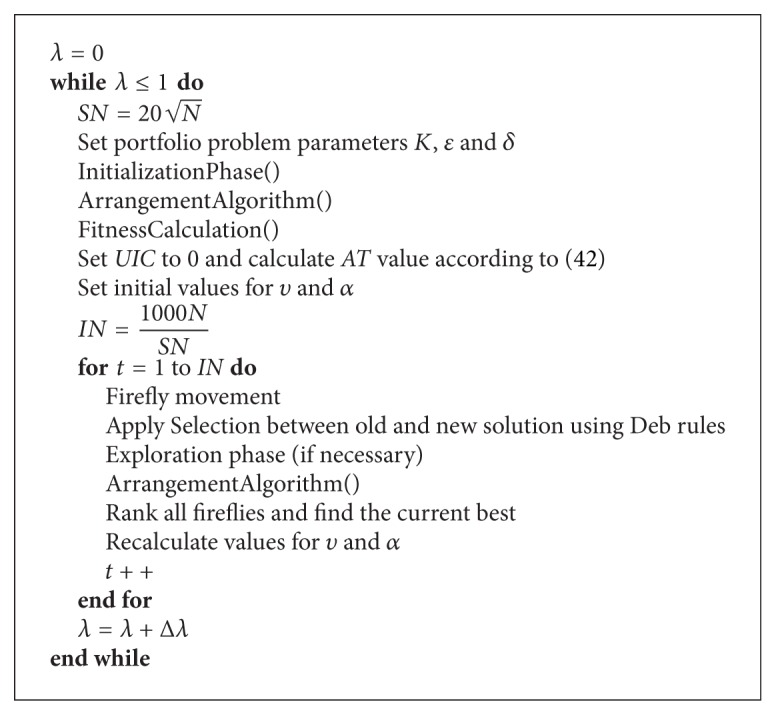
Modified firefly with parameters.

**Table 1:** Parameters.

Parameter	Value
Modified FA global control parameters	
Number of fireflies-solutions (*SN*)	Depends on *N*
Number of iterations (*IN*)	Depends on *SN*
Abandonment threshold (*AT*)	Depends on *SN* and *IN*
Exploration breakpoint (*EBP*)	Depends on *IN*

FA search parameters	
Initial value for randomization parameter *α*	0.5
Attractiveness at *r* = 0, *β*_0_	0.2
Absorption coefficient *γ*	1.0

Portfolio parameters	
Number of potential securities (*N*)	Depends on the problem
Number of assets in portfolio (*K*)	10
Initial value of risk aversion (*λ*)	0
Different *λ* values (*ξ*)	51
Lower asset's weight (*ε*)	0.01
Upper asset's weight (*δ*)	1.0
Lower bound of entropy (*L*_*E*_)	[0, ln⁡⁡*K*]

Constraint-handling parameters	
Initial violation tolerance (*υ*)	1.0
Decrement (*dec*)	1.002

**Table 2:** Benchmark specific parameters.

Parameter	Value
Hang Seng index with 31 assets	
Number of fireflies-solutions (*SN*)	111
Number of iterations (*IN*)	279
Abandonment threshold (*AT*)	3
Exploration breakpoint (*EBP*)	140

DAX 100 index with 85 assets	
Number of fireflies-solutions (*SN*)	185
Number of iterations (*IN*)	459
Abandonment threshold (*AT*)	3
Exploration breakpoint (*EBP*)	230

FTSE 100 index with 89 assets	
Number of fireflies-solutions (*SN*)	189
Number of iterations (*IN*)	479
Abandonment threshold (*AT*)	3
Exploration breakpoint (*EBP*)	240

S&P 100 index with 98 assets	
Number of fireflies-solutions (*SN*)	198
Number of iterations (*IN*)	494
Abandonment threshold (*AT*)	3
Exploration breakpoint (*EBP*)	247

Nikkei index with 225 assets	
Number of fireflies-solutions (*SN*)	300
Number of iterations (*IN*)	750
Abandonment threshold (*AT*)	3
Exploration breakpoint (*EBP*)	375

**Table 3:** Experimental results of FA and mFA for CCMV model.

Index	*N*	Performance indicators	FA	mFA
Hang Seng	31	Mean Euclidean distance	0.0006	**0.0003**
		Variance of return error (%)	1.7092	**1.2387**
		Mean return error (%)	0.7172	**0.4715**
		Execution time	**18**	20

DAX 100	85	Mean Euclidean distance	0.0032	**0.0009**
		Variance of return error (%)	7.3892	**7.2569**
		Mean return error (%)	1.4052	**1.3786**
		Execution time	**67**	71

FTSE 100	89	Mean Euclidean distance	0.0005	**0.0004**
		Variance of return error (%)	**2.6391**	2.7085
		Mean return error (%)	**0.3025**	0.3121
		Execution time	**81**	94

S&P 100	98	Mean Euclidean distance	0.0011	**0.0003**
		Variance of return error (%)	3.9829	**3.6026**
		Mean return error (%)	1.0025	**0.8993**
		Execution time	**129**	148

Nikkei	225	Mean Euclidean distance	0.0001	**0.0000**
		Variance of return error (%)	1.7834	**1.2015**
		Mean return error (%)	0.7283	**0.4892**
		Execution time	**335**	367

**Table 4:** Experimental results of mFA for CCMV model with and without entropy constraint.

Index	*N*	Performance indicators	mFA for CCMV	mFA for CCMV with entropy
Hang Seng	31	Mean Euclidean distance	0.0004	**0.0003**
		Variance of return error (%)	1.2452	**1.2387**
		Mean return error (%)	0.4897	**0.4715**
		Execution time	20	20

DAX 100	85	Mean Euclidean distance	0.0009	0.0009
		Variance of return error (%)	7.2708	**7.2569**
		Mean return error (%)	1.3801	**1.3786**
		Execution time	**70**	71

FTSE 100	89	Mean Euclidean distance	0.0004	0.0004
		Variance of return error (%)	2.7236	**2.7085**
		Mean return error (%)	0.3126	**0.3121**
		Execution time	**92**	94

S&P 100	98	Mean Euclidean distance	0.0004	**0.0003**
		Variance of return error (%)	3.6135	**3.6026**
		Mean return error (%)	0.8997	**0.8993**
		Execution time	**146**	148

Nikkei	225	Mean Euclidean distance	0.0000	0.0000
		Variance of return error (%)	**1.1927**	1.2015
		Mean return error (%)	**0.464**	0.4892
		Execution time	**360**	367

**Table 5:** Experimental results for five metaheuristics.

Index	*N*	Performance indicators	GA	TS	SA	PSO	mFA
Hang Seng	31	Mean Euclidean distance	0.0040	0.0040	0.0040	0.0049	**0.0003**
		Variance of return error (%)	1.6441	1.6578	1.6628	2.2421	**1.2387**
		Mean return error (%)	0.6072	0.6107	0.6238	0.7427	**0.4715**
		Execution time	18	9	10	34	20

DAX 100	85	Mean Euclidean distance	0.0076	0.0082	0.0078	0.0090	**0.0009**
		Variance of return error (%)	7.2180	9.0309	8.5485	**6.8588**	7.2569
		Mean return error (%)	**1.2791**	1.9078	1.2817	1.5885	1.3786
		Execution time	99	42	52	179	71

FTSE 100	89	Mean Euclidean distance	0.0020	0.0021	0.0021	0.0022	**0.0004**
		Variance of return error (%)	2.8660	4.0123	3.8205	3.0596	**2.7085**
		Mean return error (%)	0.3277	0.3298	0.3304	0.3640	**0.3121**
		Execution time	106	42	55	190	94

S&P 100	98	Mean Euclidean distance	0.0041	0.0041	0.0041	0.0052	**0.0003**
		Variance of return error (%)	**3.4802**	5.7139	5.4247	3.9136	3.6026
		Mean return error (%)	1.2258	**0.7125**	0.8416	1.4040	0.8993
		Execution time	126	51	66	214	148

Nikkei	225	Mean Euclidean distance	0.0093	0.0010	0.0010	0.0019	**0.0000**
		Variance of return error (%)	1.2056	1.2431	1.2017	2.4274	**1.2015**
		Mean return error (%)	5.3266	0.4207	**0.4126**	0.7997	0.4892
		Execution time	742	234	286	919	367
